# A Distributed-Acoustic-Sensing-Based Method for Early Warning of Urban Road Subsurface Cavities Using Vehicle-Induced Vibrations

**DOI:** 10.3390/s26206377

**Published:** 2026-10-09

**Authors:** Yidan Qin, Youliang Ding, Xiaonan Zhang, Hanwei Zhao, Shitong Hou

**Affiliations:** Key Laboratory of Concrete and Prestressed Concrete Structures of Ministry of Education, Southeast University, Nanjing 210096, China; 220241441@seu.edu.cn (Y.Q.); 230238318@seu.edu.cn (X.Z.); wudizhw_0@126.com (H.Z.); shitonghou@seu.edu.cn (S.H.)

**Keywords:** structural health monitoring, distributed acoustic sensing (DAS), urban road subsurface cavities, vehicle-induced vibrations, early anomaly monitoring

## Abstract

Rapid underground development in cities increases the hidden risks of roadbed cavities. Traditional geophysical methods, such as ground-penetrating radar (GPR), are costly and unsuitable for automated large-scale monitoring. This paper proposes a distributed-acoustic-sensing (DAS)-based algorithm for urban road cavity monitoring using vehicle-induced vibration signals. By leveraging routine urban traffic as a natural excitation source and DAS as the sensing medium, automated and continuous perception of subsurface-cavity hazards is achieved. A time–frequency joint vehicle-signal extraction algorithm is introduced to reliably isolate vehicle responses under complex noise conditions, and a physically interpretable Average Feature Ratio Indicator (AFRI) is constructed to identify long-term distribution shifts associated with structural anomalies. The AFRI remains highly stable within a narrow 0.70–1.00 range on cavity-free roads, even under low-level false positives (~1%). Experimental validation further shows that anomalous responses extend approximately 5 m from the cavity center. These findings confirm both the feasibility of the proposed method and its potential for practical early warning of subsurface cavities in urban road infrastructure.

## 1. Introduction

The rapid development of urban underground spaces has given rise to an increasingly complex subsurface infrastructure network, including drainage pipelines, gas pipelines, metro systems, and underground commercial spaces [1]. Under factors such as pipeline leakage, construction disturbances, or natural settlement, localized soil loss may occur, leading to the formation of concealed cavities. Once cavities reach a certain scale, they can trigger surface subsidence or localized collapse, posing a significant threat to roadway structural integrity and traffic safety [2,3]. Conventional geophysical methods—including GPR, laser scanning, microgravity, electrical resistivity tomography (ERT), and transient electromagnetic (TEM)—have been widely employed for subsurface anomaly identification. Their efficacy in detecting localized cavities and structural defects has been substantiated across multiple studies [4,5,6]. However, these methods predominantly rely on discrete point sampling and manual deployment. As a result, they suffer from limited operational efficiency, restricted spatial coverage, and high implementation costs [7,8]. To improve detection, researchers have explored the development of vehicle-mounted mobile sensing systems [9] and strategies for optimizing observation-point deployment [10]. These improvements have achieved partial success in localized applications but remain fundamentally insufficient for long-term, automated early-warning systems for cavities under urban roads.

DAS enables continuous monitoring of stress and strain along fiber optic cables by detecting phase changes in backscattered light [11,12]. Compared with conventional point sensors, DAS offers high spatiotemporal resolution, long-range coverage, and ease of deployment. Existing urban fiber-optic networks can be directly repurposed for DAS, allowing large-scale, continuous monitoring of urban roadways [13]. Previous studies have employed DAS to invert shallow-subsurface wave-velocity structures [14] and image the distribution of urban underground layers [15]. These studies demonstrate that DAS can capture the propagation characteristics of surface wavefields and further characterizes heterogeneity in near-surface local structures. As a result, it provides a high-resolution data foundation for subsurface-cavity detection. Moreover, DAS exhibits high sensitivity to structural damage and localized anomalies across a range of engineering scenarios [16,17,18,19,20]. Tan et al. (2021) implemented Optical Frequency Domain Reflectometry (OFDR)-based fiber sensors on steel pipes and detected corrosion through real-time distributed strain measurements [21]. Liu et al. (2023) turned existing telecommunication fiber-optic cables into distributed acoustic sensors for vibration-based bridge health monitoring [22]. Wang et al. (2024) monitored train operations with optical fibers, identifying track anomalies along a 12 km segment of the Beijing–Guangzhou High-Speed Railway [23]. DAS has shown great promise in revealing subsurface soil dynamics and detecting structural anomalies, making it a powerful tool for early detection of concealed cavities. It has been preliminarily explored in recent studies [24,25,26].

Nevertheless, existing DAS-based anomaly monitoring largely relies on stable, high-intensity external excitations, such as high-speed trains, pipeline flows, or artificially imposed signals. In urban road environments, such excitations are generally unavailable. Artificially applying excitations is not only costly but also difficult to implement for large-scale, real-time monitoring. In contrast, the proposed framework utilizes urban traffic flow as a natural and sustainable source of vehicle-induced vibrations, avoiding the need for externally controlled excitation. In recent years, numerous studies have confirmed the effectiveness of DAS in sensing signals related to traffic [27,28,29]. For example, He et al. (2024) and Liu et al. (2025) respectively applied DAS in the Chongqing Integrated Utility Tunnel and along San Jose Road. They achieved real-time identification and dynamic reconstruction of traffic loads, pedestrian activities, and construction disturbances [30,31]. The results confirm that DAS effectively senses traffic-induced surface vibrations. This finding highlights its potential for leveraging urban traffic excitations in monitoring subsurface-cavity anomalies.

Despite the demonstrated potential of DAS in previous studies, its implementation in urban road scenarios still faces critical challenges. Firstly, traffic-induced excitations are highly random and nonstationary, making it difficult to effectively separate vehicle responses from environmental noise. Secondly, machine learning and deep learning approaches have been incorporated into DAS data processing for noise suppression and complex signal pattern recognition [32,33,34,35]. However, these methods rely heavily on large annotated datasets, offer limited physical interpretability, and demand substantial computational resource [36,37]. These limitations hinder the practical deployment of DAS for cost-effective, long-term, and automated urban road health monitoring.

To overcome these challenges, this study proposes a DAS-based framework for urban road subsurface cavity detection driven by vehicular excitations. Unlike approaches that depend on controlled or relatively stable excitation sources, the framework leverages routine traffic flow as a natural and sustainable excitation source and utilizes existing telecommunication optical fibers to establish a cost-effective and continuous road health-monitoring platform. The major contributions of this work are summarized as follows:

(1) A time–frequency joint vehicle-signal extraction algorithm is developed to effectively isolate vehicle-induced vibrations under highly nonstationary excitation conditions.
(2) A physically interpretable anomaly index is constructed based on time and frequency feature ratios, enabling cavity anomaly detection under limited labeled data conditions.
(3) Multi-scenario experiments, including controlled test roads and in-service urban roads, are conducted to validate the framework. The results demonstrate low-error identification of potential cavities and confirm the framework’s feasibility for long-term automated monitoring of urban road integrity.

## 2. Methods

### 2.1. Mechanism Overview

**Figure 1:**
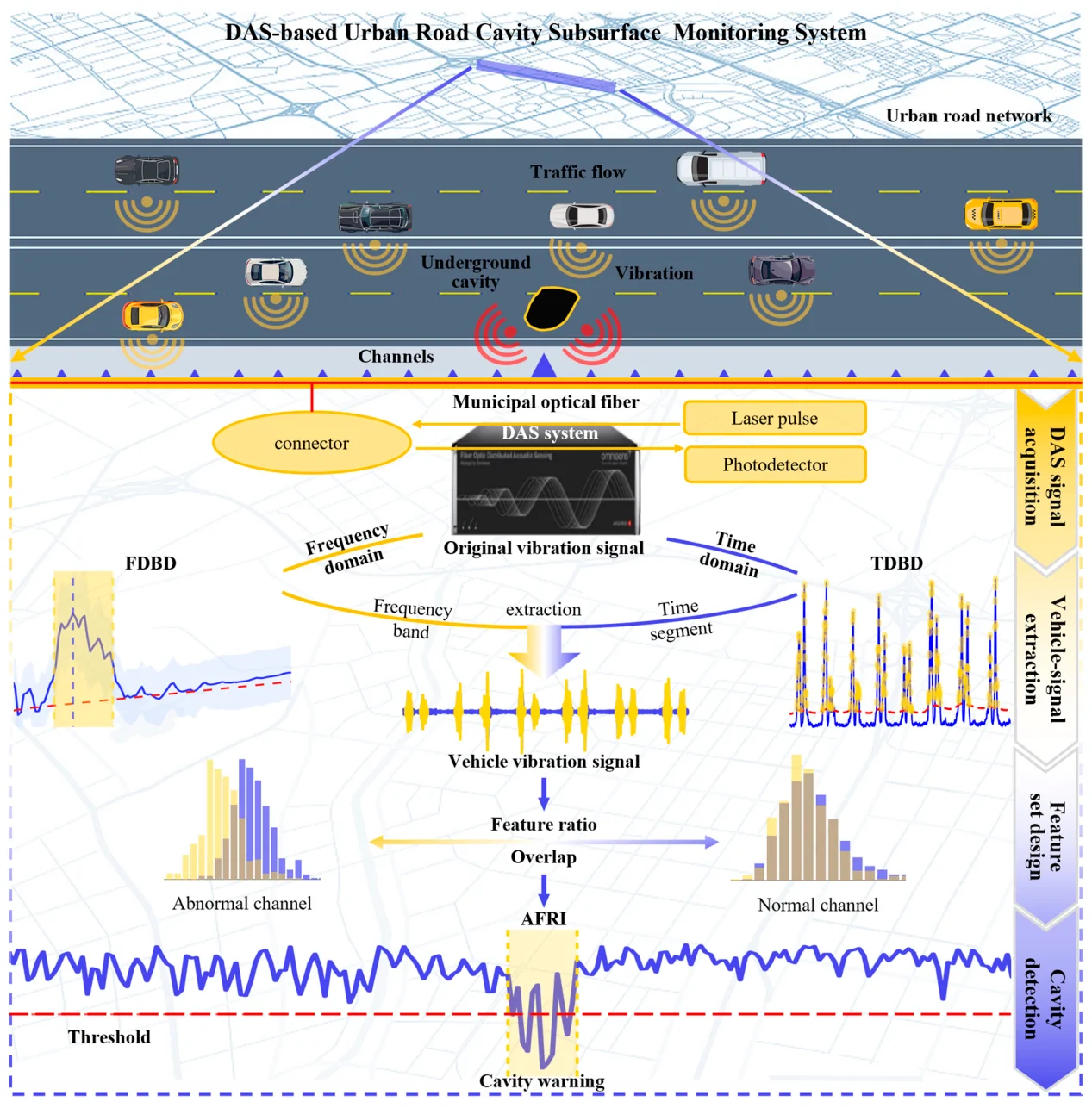
Technical roadmap of the DAS-based urban road subsurface-cavity-monitoring system.

Figure 1 illustrates the overall workflow of the proposed DAS-based urban road subsurface-cavity-monitoring framework. The framework comprises four modules: (1) DAS signal acquisition: The existing subsurface optical fibers beneath urban roadways are connected to DAS interrogators to acquire and decode vibration signals under complex environmental excitations. (2) Vehicle-signal extraction: Vehicle-induced vibration components are dynamically extracted using a joint time–frequency approach. This method first adaptively identifies dominant vehicular frequency bands in the frequency domain to suppress background noise. Subsequently, dynamic thresholding and connectivity analysis are applied in the time domain to robustly extract vehicle response segments. (3) Feature set design: A set of feature ratios exhibiting high sensitivity and stability to cavity-related structural responses is selected from candidate time–frequency features. (4) Cavity detection: An overlap-based statistical index is constructed to quantify anomalies, and potential subsurface-cavity regions are identified through an adaptive thresholding scheme.

### 2.2. Principle of DAS

The DAS system used in this study operates on the principle of coherent Rayleigh scattering, enabling high-resolution vibration sensing along the entire fiber length. Broadband swept-frequency optical pulses generated by a laser are injected into the sensing fiber after optical modulation and amplification. When the fiber experiences external vibrations, the photoelastic effect causes minute changes in its length and local refractive index, which modulate the phase and intensity of the backscattered light. The coherent receiver captures the backscattered signals, which are then decoded through high-speed data acquisition and processing to reconstruct the temporal and spatial vibration distributions along the fiber. This enables accurate recording of both vehicle-induced and environmental structural responses.

### 2.3. Extraction of Vehicle-Induced Vibration Response Signals

After demodulation, the DAS vibration signal forms a 2-D spatiotemporal matrix, whose channels are uniformly distributed along the fiber. The signal consists of vehicle-induced excitations, environmental disturbances, and system noise, and it cannot be directly used for cavity detection. Robustly isolating the vehicle-dominated dynamic response from this complex background is therefore the key challenge. To address this challenge, we propose a joint time–frequency processing method that exploits both spectral and instantaneous features. This approach enables reliable extraction of vehicle-dominated responses with clear physical interpretability.

#### 2.3.1. Frequency-Domain Detrended Burst Detection

Vibration signals collected from urban road optical fibers exhibit a low signal-to-noise ratio and complex background noise. As a result, vehicle-induced pavement vibrations are difficult to identify directly in the time domain. However, frequency-domain analysis can highlight localized energy peaks.

**Figure 2:**
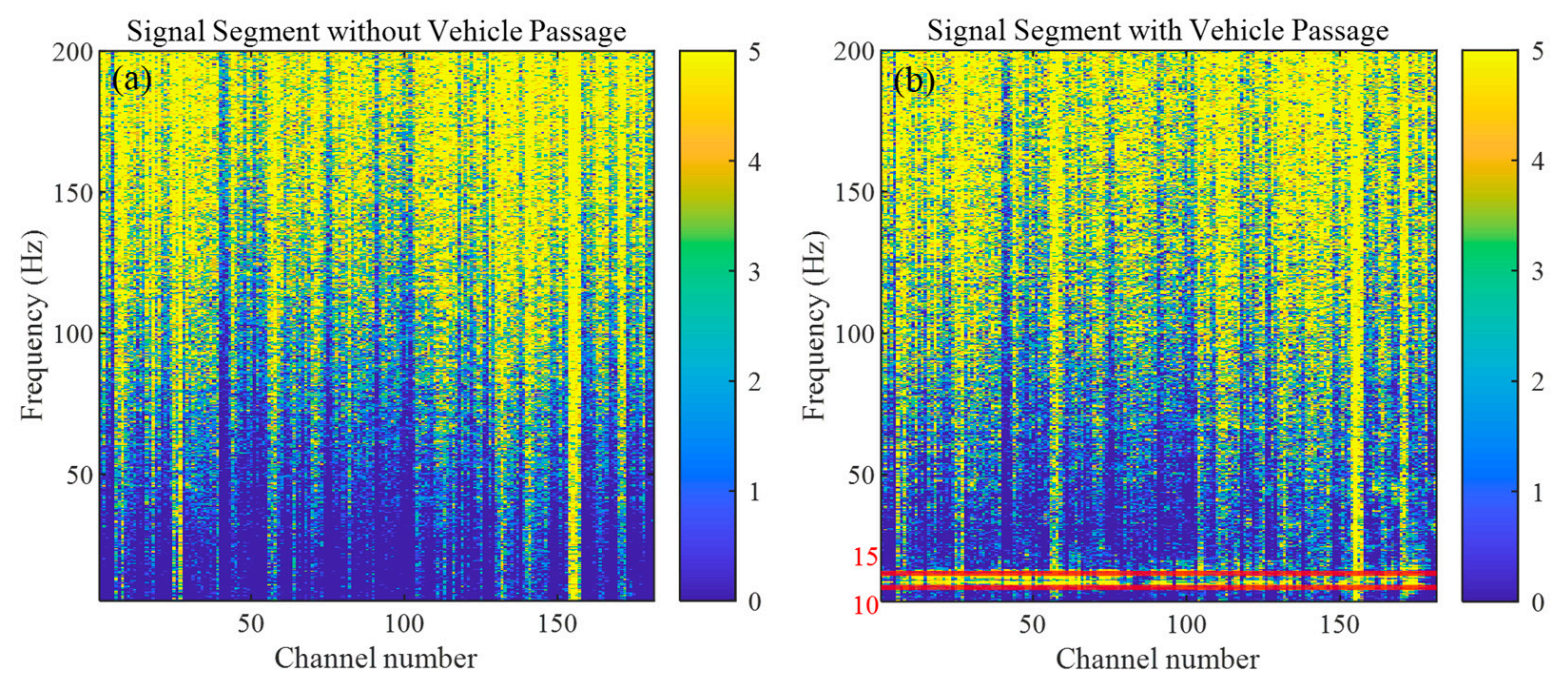
Frequency–channel distribution: (**a**) signal segment without vehicle passage, (**b**) signal segment with vehicle passage.

Figure 2a,b show frequency–channel maps of vibration signals with and without vehicles. Each channel exhibits complex background noise. During vehicle passages, high-amplitude frequency components emerge across channels compared to the no-vehicle condition, particularly in the 10–15 Hz range (Figure 2b). This indicates that vehicle-induced response is primarily concentrated within localized frequency bands. Effective extraction therefore requires removing the background trend in advance.

Accordingly, this paper introduces the Frequency-Domain Detrended Burst Detection (FDBD) algorithm based on frequency-domain characteristics. Vehicle-dominant vibrational energy is primarily concentrated within the 5–100 Hz range. This range was selected based on preliminary spectral observations of the measured DAS signals and the physical characteristics of the recorded vehicle-induced vibrations. Signals below 5 Hz mainly reflect overall road settlement and slow vehicle sway, while components above 100 Hz are predominantly environmental noise and transient impulses. Therefore, the analysis frequency is confined to 5–100 Hz to cover the main energy range of vehicle-induced vibrations while minimizing significant interference. The dominant frequency band within this range was then adaptively identified using the FDBD procedure.

To reduce amplitude discrepancies among channels and emphasize consistent frequency features, the time-series signal of each channel was first processed using the Welch segment-averaging method. The resulting spectra were then averaged across all channels to obtain the multi-channel logarithmic spectrum $\bar{P_{dB}}[k]$. Next, the background trend of the average logarithmic power spectrum was fitted using a weighted least-squares method with smoothing regularization. The optimization objective for estimating the baseline B[k] is formulated as:

(1) $$B=arg\;\underset{B}{min}\sum_{k=1}^{N}w_{k}{\left(\bar{P_{dB}}[k]-B[k]\right)}^{2}\;+\lambda \sum_{k=2}^{N}{\left(\Delta^{2}B[k]\right)}^{2}\;,$$

Here, $\Delta^{2}B[k]=B[k+1]-2B[k]+B[k-1]$ represents the second-order difference for smoothing, and the asymmetric weights $w_{k}$ ensure that the baseline follows the lower envelope of the spectrum. The parameter λ controls the degree of smoothness. This method requires no prior assumptions about frequency content or spectral shape, allowing adaptive separation of the overall spectral trend from localized energy peaks.

After subtracting the baseline, the residual spectrum $\tilde{P_{dB}}[k]$ with enhanced localized energy peaks is obtained. A sliding window $\Omega_{j}$ with width W and step size Δf is then applied to the residual spectrum to compute the windowed energy:

(2) $$\;E_{j}=\sum_{k\in \Omega_{j}}{\left(\tilde{P_{dB}}[k]\right)}^{2},$$

The window $\Omega^{*}$ exhibiting the maximum energy is selected as the vehicle-dominated frequency band. Finally, a band-pass filter within this range is applied to the original signal, highlighting vehicle-dominated vibrations while suppressing background frequency noise.

#### 2.3.2. Time-Domain Dynamic Burst Detection

After applying a band-pass filter to isolate the vehicle-dominated low-frequency response, it remains necessary to identify the time intervals corresponding to vehicle-induced vibrations. To achieve this, a Time-Domain Dynamic Burst Detection (TDBD) algorithm is proposed.

**Figure 3:**
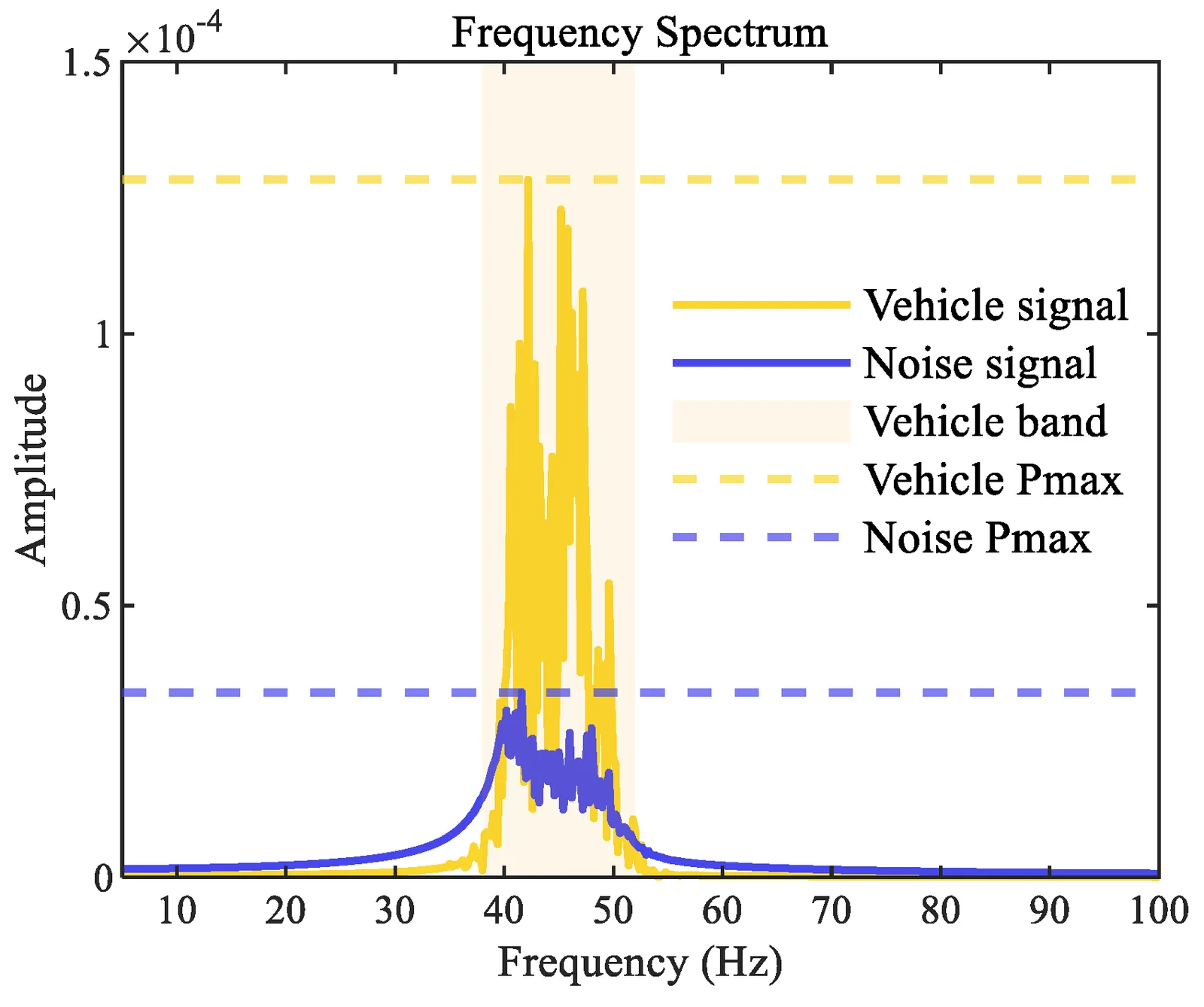
Comparison of frequency amplitudes between vehicle-induced and noise signal windows.

First, short-time Fourier transform (STFT) is applied to the filtered signal. Within each time window, the maximum spectral amplitude is extracted to form a feature sequence. As illustrated in Figure 3, the maximum spectral amplitudes differ between vehicle-induced and noise-only signal windows, demonstrating the potential of this feature for distinguishing vehicle excitations from background noise. Unlike spectral mean or total energy metrics, the maximum spectral amplitude highlights transient, high-energy events within each window, leading to more reliable vehicle-passage detection under low-signal-to-noise conditions.

In real road scenarios, variations in vehicle type, speed fluctuations, and pavement conditions introduce strong non-stationarity, making a fixed threshold ineffective. Therefore, the TDBD method employs a dynamic background modeling strategy to adaptively determine detection thresholds.

In the initial phase, a quantile filter is used to suppress sporadic high-energy anomalies, allowing robust estimation of the background mean $\mu_{init}$ and standard deviation $\sigma_{init}$. The length of this initial segment must be moderate to avoid overestimation of the background due to dominant vehicle-induced signals. To capture temporal variations in noise, μ(n) and σ(n) are continuously updated using statistics from preceding and subsequent windows. Accordingly, the background statistics and detection threshold are determined adaptively from the measured signal rather than being fixed a priori.

The adaptive threshold for the current window θ(n) is then defined as follows:

(3) $$\mu (n)=(1-\alpha )\mu_{\mathrm{prev}}+\alpha \mu_{\mathrm{future}},$$

(4) $$\sigma (n)=(1-\alpha )\sigma_{\mathrm{prev}}+\alpha \sigma_{\mathrm{future}},$$

(5) θ(n)=μ(n)+βσ(n),

When $P_{max}(n)>\theta (n)$, the corresponding window is identified as a vehicle-excitation segment. Connected-component analysis is then applied to eliminate short-term false positives, and adjacent valid windows are merged into continuous segments. These segments are subsequently partitioned into equal-length output intervals. By utilizing adaptive thresholding, the TDBD algorithm can robustly and automatically extract vehicle-dominated vibration segments under varying traffic conditions and pavement environments.

In summary, Section 2.3.1 and Section 2.3.2 integrate frequency- and time-domain processing to reliably extract vehicle-dominated vibrations. The resulting signals serve as robust inputs for constructing cavity-sensitive indicators derived from local vibration features.

### 2.4. Construction of Frequency–Time-Domain Feature Metrics

In this study we construct a set of indicators sensitive to local structural anomalies from time–frequency features to characterize vehicle-dominated vibrations, enabling quantitative subsurface-cavity monitoring. First, correlation analysis is performed on a large pool of candidate time–frequency features to eliminate redundant indicators. Subsequently, mutually independent feature metrics are selected.

Given the high spatial resolution of the DAS system, adjacent measurement points experience approximately the same excitation. Under this assumption, feature ratios were introduced to reduce uncertainty caused by variations in excitation amplitude and to amplify vibration anomalies associated with local damage. This approach enhances both the stability and the physical interpretability of the state indicators. The feature ratio of the *i*-th feature in the *n*-th channel is defined as:

(6) $$R_{i,n}=\frac{F_{i,n}}{(F_{i,n-1}+F_{i,n}+1)/2},$$

**Figure 4:**
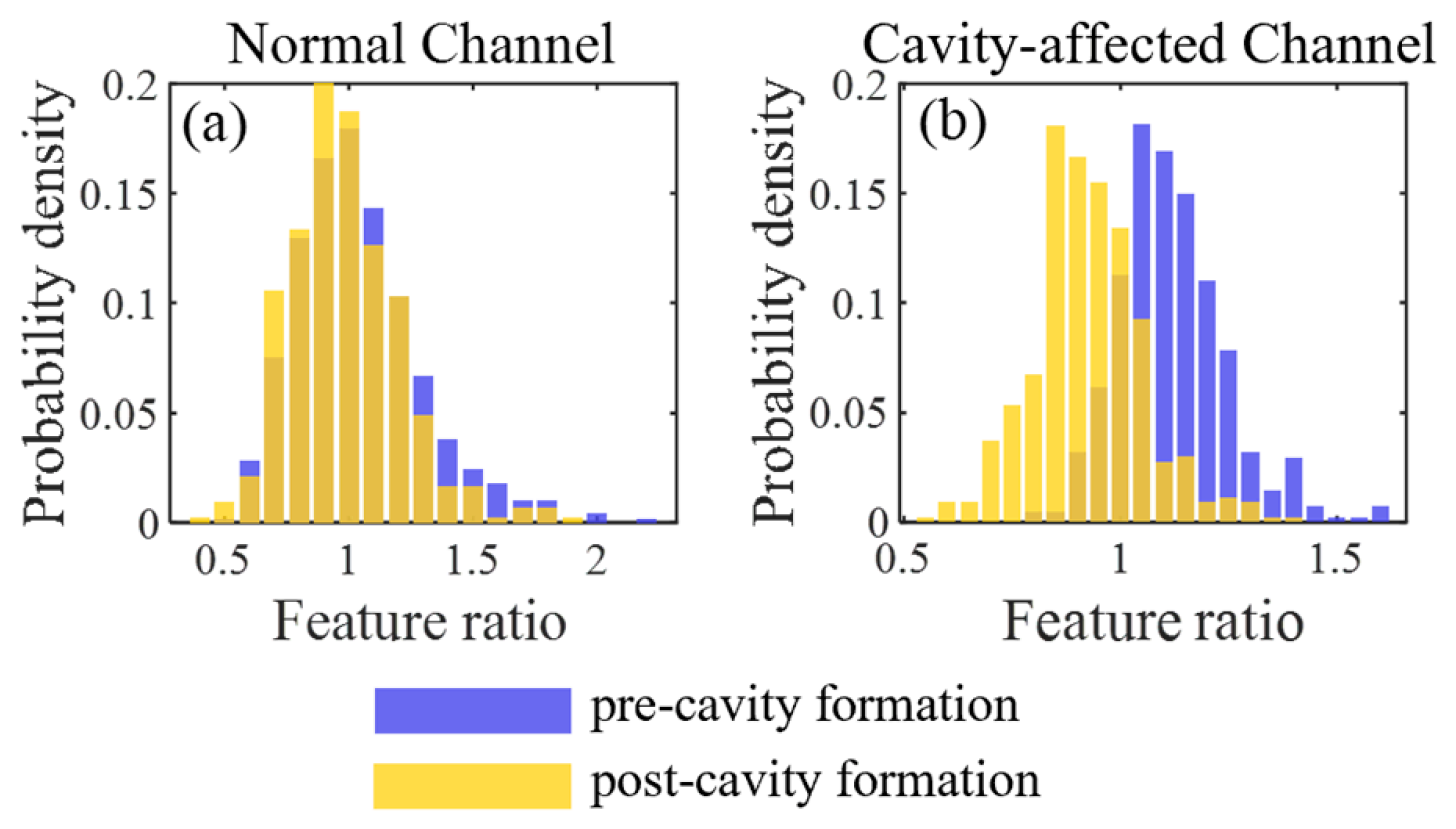
Feature ratio statistical distributions: (**a**) normal channel, (**b**) cavity-affected channel.

As shown in Figure 4, the feature ratio distributions of vehicle-induced signal samples remain largely consistent across normal road sections, reflecting stable subsurface conditions. In contrast, channels corresponding to cavity regions exhibit a pronounced distribution shift, indicating significant subsurface structural changes. These differences demonstrate that the feature ratio distributions are sensitive to variations in vibration energy and frequency structure induced by cavities.

To quantify the discriminative ability of each feature ratio, an overlap metric is introduced. The overlap $\omega_{i}$ is defined as the proportion of paired data points between two feature ratio datasets $R_{i}^{\left(1\right)},R_{i}^{\left(2\right)}$ within a tolerance e, normalized by the size of the smaller dataset. Mathematically, it is expressed as:

(7) $$\omega_{i}=\frac{I(R_{i}^{\left(1\right)},R_{i}^{\left(2\right)})}{\min(N_{i}^{\left(1\right)},N_{i}^{\left(2\right)})},$$

Based on the aforementioned analysis, the distribution overlap degree of each feature ratio across all channels can be statistically evaluated. Feature ratios with lower overall overlap are considered more sensitive to cavity-related anomalies and are selected to construct an optimal feature ratio set for cavity discrimination.

### 2.5. Road Anomaly Assessment Based on Average Feature Ratio Indicator

To quantitatively identify local subgrade anomalies, an AFRI is developed based on the $N_{f}$ selected optimal feature ratios. For the n-th channel, each feature ratio is calculated under two batches of Figure data, denoted as $R_{i}^{\left(1\right)}$ and $R_{i}^{\left(2\right)}$, and the overlap $\omega_{i,n}$ between the two sets is computed. The average overlap across all features is then taken as the comprehensive index for the channel:

(8) $$I_{n}=\frac{1}{N_{f}}\sum_{i=1}^{N_{f}}\omega_{i,n},$$

In the process of implementing quantitative AFRI-based discrimination, statistical modeling is first conducted on indicator samples from cavity-free conditions. The AFRI follows a right-skewed distribution with convergent mean and variance characteristics. Therefore, the threshold is determined based on the convergence principle, rather than fixed quantiles derived from normal-distribution assumptions. The resulting lower-bound threshold must effectively cover the fluctuations of normal channels while maintaining a low false-positive rate, providing a robust baseline for anomaly detection. A newly measured AFRI value below the threshold flags the corresponding channel as a potential cavity anomaly, enabling quantitative identification and early warning of subgrade defects.

## 3. Results

### 3.1. Sensitivity Testing in Experimental Road

#### 3.1.1. Experimental Road System Configuration

**Figure 5:**
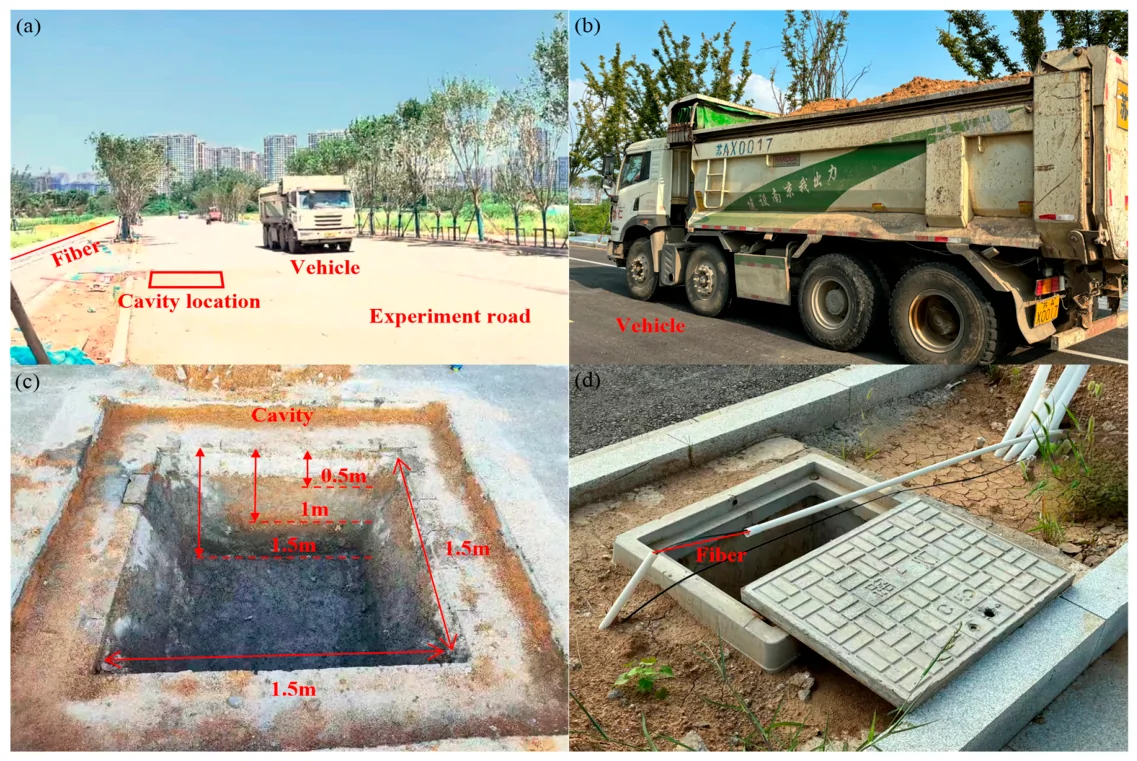
Schematic of the experimental site layout and test conditions: (**a**) overall layout of the experimental site, (**b**) four-axle truck used for vehicle excitation, (**c**) cavity horizontal dimensions and three burial depth conditions, (**d**) schematic of fiber installation.

Considering the safety risks and management constraints of excavating cavities on operational urban roads, a controlled experiment was conducted on a closed road section. The test site was a roughly 100 m long urban road in Nanjing, Jiangsu Province, China, consisting of loess soil covered by an asphalt layer. A municipal-standard single-mode optical fiber served as the distributed sensor. The 70.4 m fiber was buried in a 1 m deep Polyvinyl Chloride (PVC) conduit along the outer side of the non-motorized lane. The system operated at a 4 kHz sampling frequency with a spatial resolution of 0.8 m. To create controllable subsurface anomalies, a square cavity with dimensions of 1.5 m × 1.5 m was excavated manually. The burial depth was set to 0.5 m, 1.0 m, and 1.5 m to form a series of conditions with increasing depth. Vehicle excitation was provided by a four-axle truck weighing approximately 20 t, traveling back and forth at 20 km/h to simulate intermittent traffic. The overall experimental layout, excitation vehicle, cavity dimensions and burial depth conditions, and fiber installation configuration are shown in Figure 5.

Under each condition, the DAS system acquired a 2-D vibration data matrix. The spatial dimension consisted of 71 channels uniformly distributed along the fiber, with channel 12 corresponding to the cavity center. The temporal dimension contained approximately 14.4 million samples, representing one hour of continuous acquisition at 4 kHz. This setup enabled continuous monitoring of local anomalies along the road and provided a comparable basis for analyzing response differences under varying cavity depths.

#### 3.1.2. Vehicle-Induced Signal Extraction

**Figure 6:**
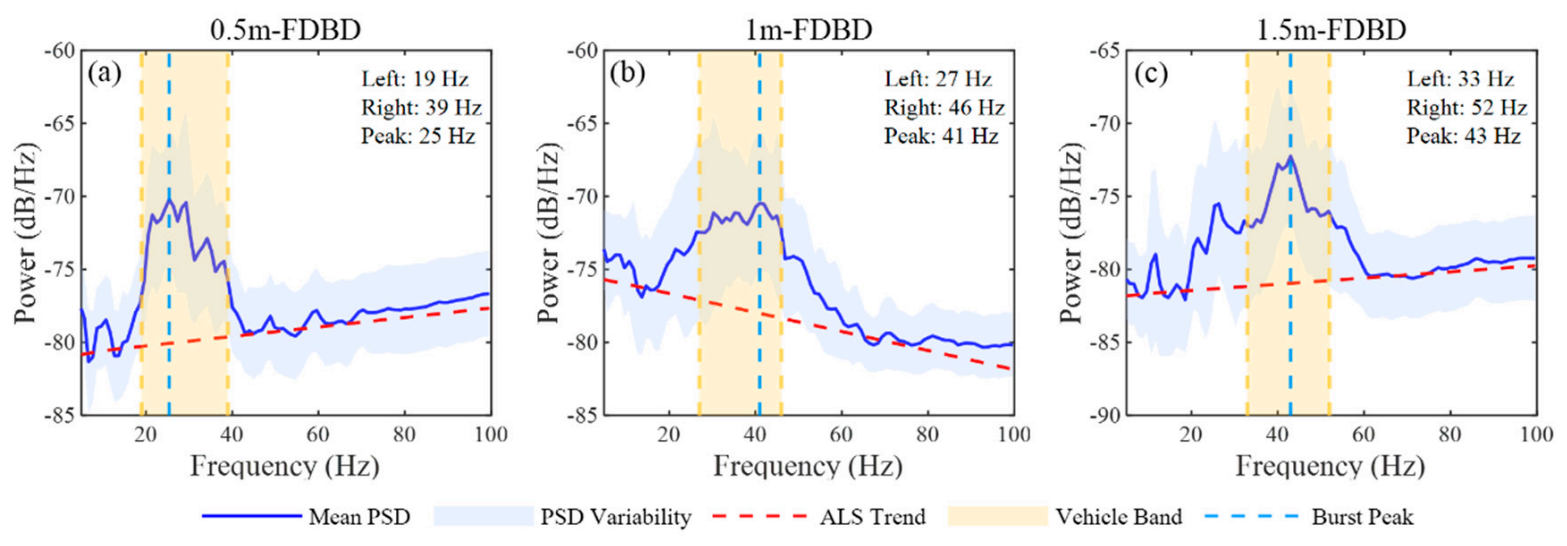
FDBD-based vehicle-induced frequency band selection for cavities of 0.5, 1, and 1.5 m depths (**a**–**c**). The yellow shaded area represents the vehicle band, with yellow dashed lines marking its lower and upper frequency boundaries.

The raw data from the three conditions were analyzed in the frequency domain using the FDBD method. A weighted least squares approach was first employed to fit the baseline of the average logarithmic power spectrum. The residual spectrum obtained after baseline removal represented the effective noise-free response. A 20 Hz sliding window was then applied to identify the frequency band with the highest signal-to-noise ratio. Figure 6 shows a consistent shift in the local energy peaks toward higher frequencies with increasing cavity depth. This pattern indicates that subsurface structural changes produce measurable variations in the frequency response.

**Figure 7:**
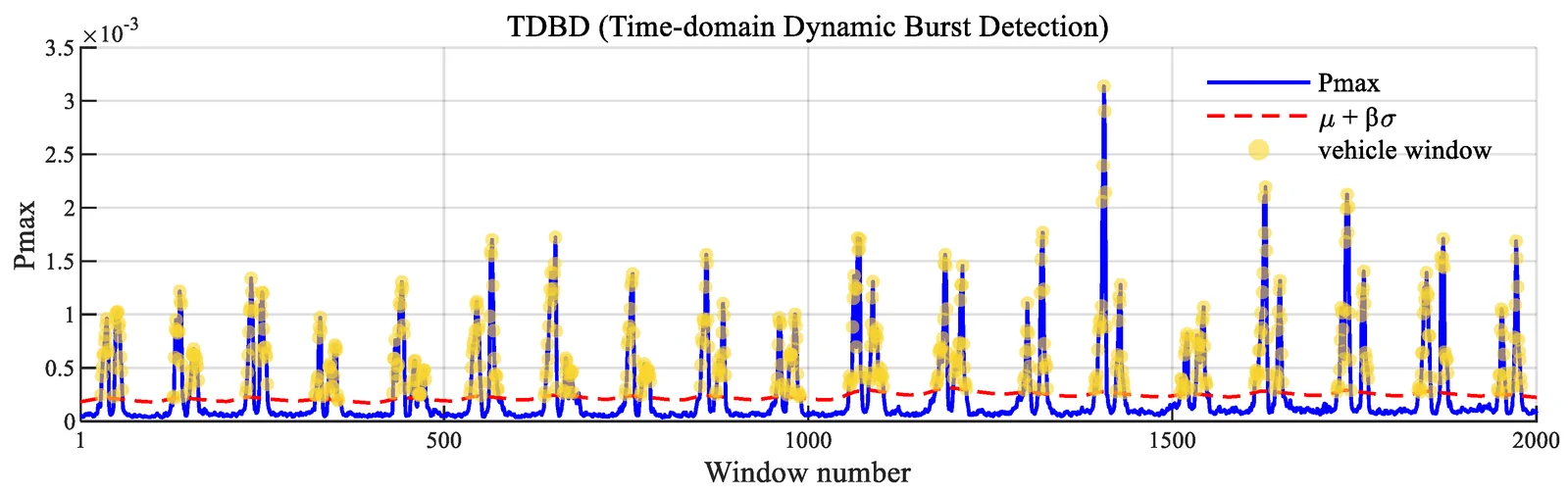
TDBD-based extraction of vehicle-induced signal windows for a single channel.

Building on these results, vehicle-induced signal segments were further extracted in the time domain using the TDBD method. Figure 7 illustrates the process of dynamic threshold determination and vehicle-induced signal window identification. The global background noise level was first estimated from the low quantile of maximum spectral amplitudes in the initial time windows. The background threshold for each window was then adaptively updated using both preceding and subsequent windows. Because the background noise remained stable throughout the experiment, the estimated thresholds also exhibited consistent behavior. Windows whose maximum spectral amplitude exceeded the threshold were identified as vehicle-induced signal segments.

**Figure 8:**
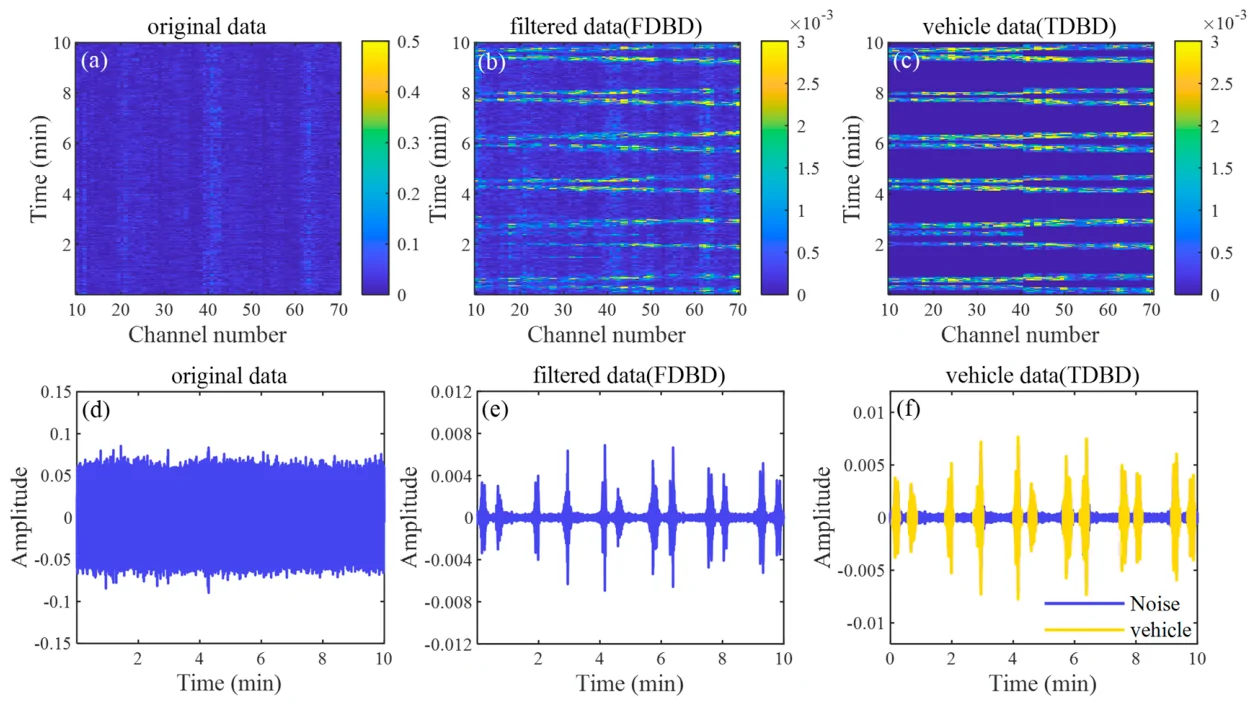
Spatiotemporal waterfall plots and time-domain waveforms: (**a**,**d**) original data, (**b**,**e**) filtered data after FDBD, (**c**,**f**) vehicle-induced data after TDBD.

As shown in Figure 8, the original vibration data were heavily contaminated by complex background noise, making vehicle-induced signatures almost indistinguishable in both waterfall and time-domain views. After joint time–frequency denoising, clearly vehicle-induced signals emerged. The waterfall plot reveals significant suppression of background noise, with sharp and continuous signal boundaries. The waterfall plot shows strong suppression of background noise and distinct, continuous signal boundaries. In the time domain, the signals appeared at regular intervals consistent with the vehicle excitation frequency. Spatially, adjacent channels exhibited synchronized temporal responses, further confirming the physical validity of the extraction method.

#### 3.1.3. Construction of the Effective Feature Set

Feature ratios for each channel were first computed at high spatial resolution to minimize the influence of external excitation variability. Redundancy screening was then performed through correlation analysis. When the Pearson correlation coefficient between two feature ratios exceeded the preset threshold (|r| > 0.85), only the feature with a stronger association with cavity identification was retained. This process reduced inter-feature redundancy. After screening, twenty feature ratios with low inter-correlation and complementary information were preserved, as summarized in Table 1 and Table 2.

Based on datasets corresponding to different cavity depths, the cumulative overlap of each feature ratio across all channels was calculated. Feature ratios with lower overlap proved to be more sensitive to structural anomalies. Finally, five of these were selected to construct the optimal feature set. The selected features—spectral kurtosis, spectral skewness, spectral amplitude factor, spectral waveform factor, and spectral crest factor—are all derived from frequency-domain indicators. These indicators describe the signal’s amplitude impulsiveness, spectral sharpness, and morphological distribution. This combination of them allows for the capture of local dynamic alterations in the vehicle-induced vibration response from subsurface cavities.

#### 3.1.4. Setting of Alarm Thresholds

Based on the five optimal feature ratios with physical interpretability, the AFRI was calculated by comparing the feature ratios of the same channel under two distinct operational conditions. This index quantitatively characterizes localized anomalies along the sensing fiber. For multiple operational conditions, pairwise comparative analyses were sequentially performed to obtain AFRI values for every channel, allowing cross-condition assessment of anomaly sensitivity.

**Figure 9:**
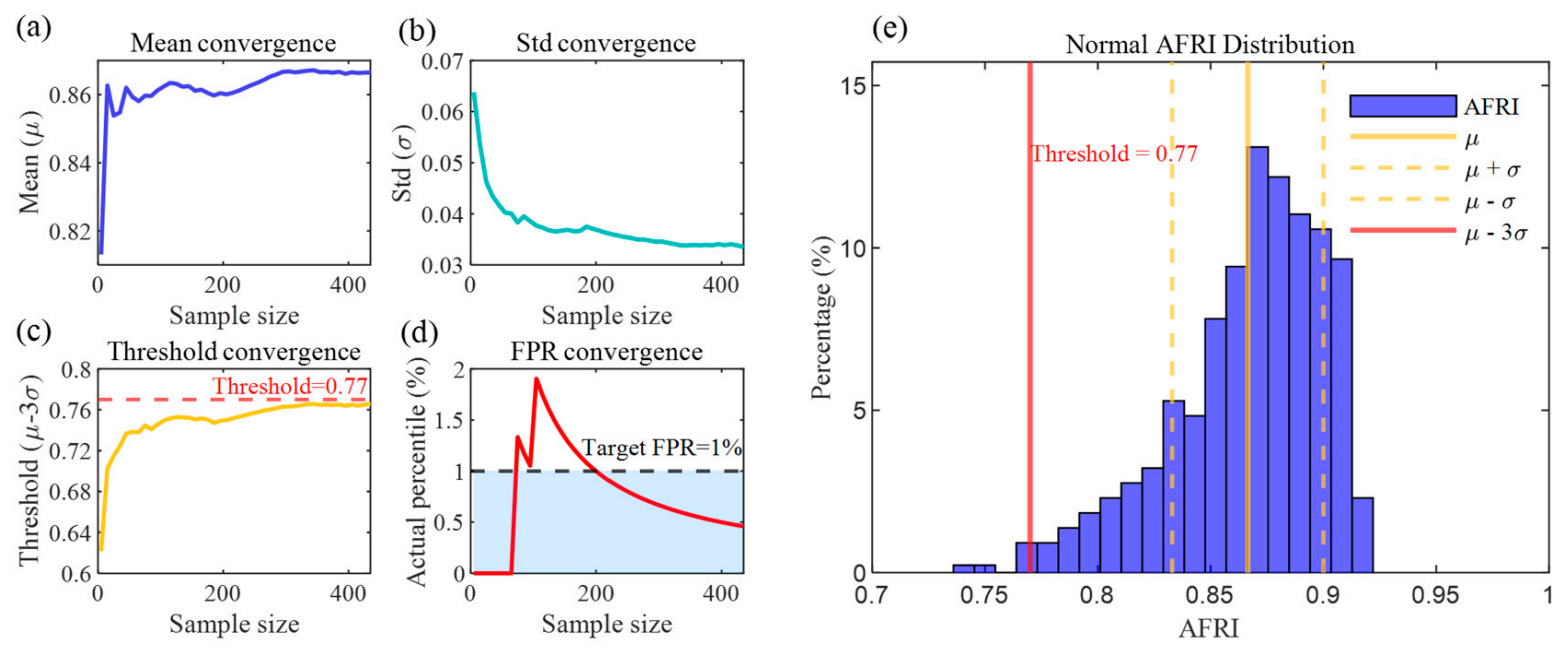
AFRI distribution and threshold analysis under normal conditions: (**a**) mean convergence, (**b**) standard deviation convergence, (**c**) threshold convergence, (**d**) actual false-positive-rate (FPR) convergence, (**e**) normal AFRI distribution.

To further establish a unified decision criterion, AFRI was calculated using data from the same channel under the same operating condition. These data reflected a consistent subsurface state and therefore describe the AFRI distribution under normal conditions. This characterization served as a reliable reference for threshold determination. As illustrated in Figure 9, with a sample size of 400, AFRI values ranged between 0.7 and 1.0 and exhibited a right-skewed pattern. As the sample size increased, both the mean and variance gradually converged, resulting in a stable distribution.

Targeting at approximately a 1% false positive rate, the lower threshold for AFRI was established based on the convergence behavior of the mean and standard deviation. Since the AFRI distribution was markedly right-skewed, the 1% quantile under a normal distribution μ−2.33σ could not be directly applied for threshold determination. Instead, the lower statistical bound was defined as *μ − 3σ*, and its convergence was monitored as the sample size increased. When the sample size exceeded 300, the *μ − 3σ* value statistically stabilized and converged to 0.77. The actual lower-tail percentile corresponding to this threshold was then evaluated, and it remained below 1%. This result demonstrates that adopting 0.77 as the lower threshold satisfies both the statistical convergence criterion and the target false-positive-rate constraint.

#### 3.1.5. Analysis of Cavity Anomaly Results

**Figure 10:**
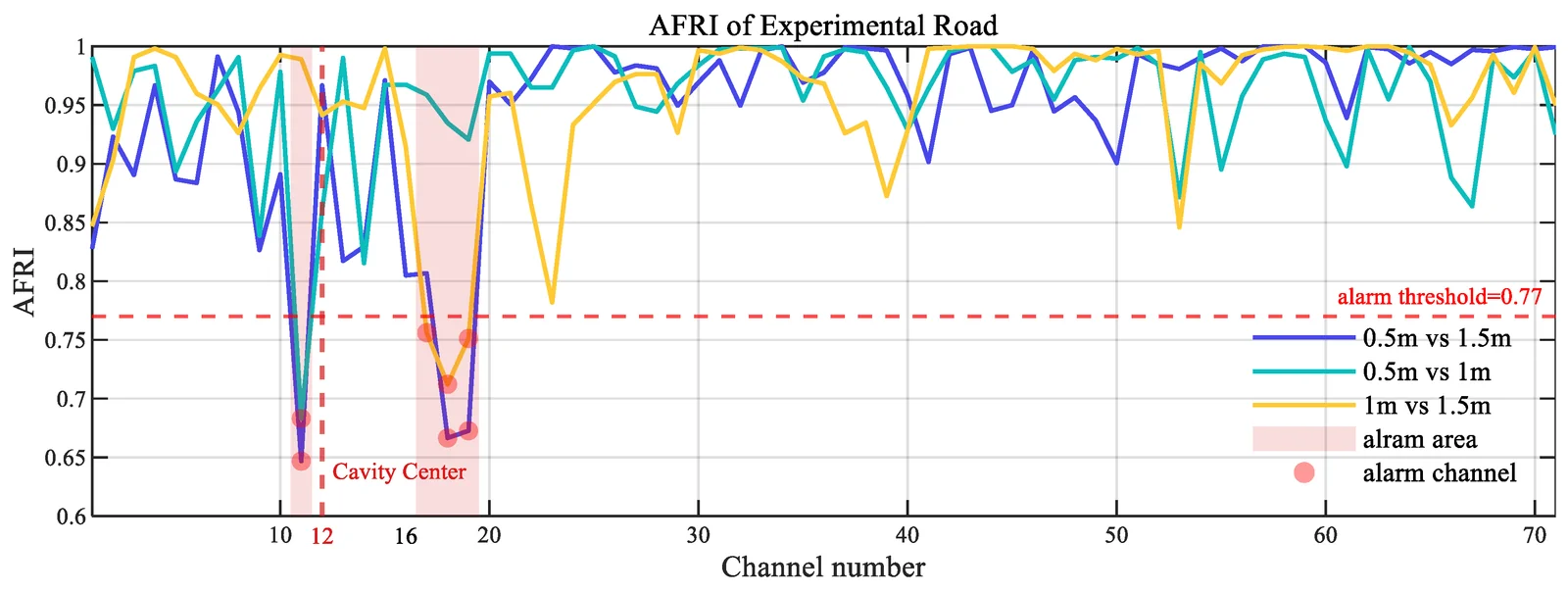
AFRI comparison across three cavity depths and corresponding alarm regions.

Three sets of full-channel AFRI results were obtained through pairwise comparisons of three working conditions with different cavity depths, evaluated using the threshold μ−3σ=0.77. As shown in Figure 10, channel 12 corresponds to the geometric center of the cavity. Channel 11 and Channels 17–19 exhibit AFRI values well below 0.77, indicating pronounced low-overlap responses. These anomalous responses were observed within approximately 5 m of the cavity center. In contrast, AFRI values in the remaining channels, despite some fluctuations, remain above the threshold. Comparing conditions with larger depth differences reveals stronger AFRI deviations. In particular, the 0.5 m vs. 1.5 m comparison (depth difference 1.0 m) produced more and larger below-threshold AFRI values than the 0.5 m vs. 1.0 m and 1.0 m vs. 1.5 m comparisons. This pattern indicates that larger subsurface changes produce clearer anomalous signatures. Overall, the results demonstrate that AFRI can reliably separate anomalous from normal sections and is sensitive to road cavities and related structural defects.

Notably, the cavity-induced AFRI anomalies are not confined to the cavity itself but extend into the surrounding area, forming a distinct spatial expansion pattern. The underlying mechanism can be interpreted from three perspectives: First, the cavity alters local boundary conditions, causing the soil to develop localized modes and standing waves within specific frequency bands. Incident waves undergo reflection, refraction, and scattering at the cavity interface, subsequently superimposing with direct waves to form complex phase interference. This phenomenon shifts energy peaks away from the center and promotes their diffusion to surrounding areas [38]. Second, the optical fiber records the integrated dynamic response of neighboring soil. The flexible coupling and soil heterogeneity lead to the redistribution of vibration energy around the cavity [39]. Additionally, continuous vehicle excitation further performs dynamic frequency–position scanning of the wavefield, amplifying spatial diffusion and offset of anomalous features [40]. However, such spatial shifts do not compromise early-warning capabilities. By narrowing the suspected region, effective detection of road defects and timely risk alerts can still be achieved.

### 3.2. Field Stability Test on Actual Roads

#### 3.2.1. Actual Road System

Although the previous experiments have verified the sensitivity of the proposed method to void detection, its stability still required further evaluation. Specifically, we further assessed the stability of the time–frequency vehicle-induced signal extraction method and the AFRI index. To do so, vehicle-induced vibration data were collected from two road sections in Jiangsu Province, China, on different dates and at multiple times. The datasets include diverse vehicle types, large traffic fluctuations, and complex temporal distributions. Data from Road 1 were collected on two consecutive sunny days, covering both daytime and nighttime traffic. And Road 2 was recorded over three consecutive days—the first two sunny and the third rainy—also encompassing multiple time periods. GPR inspection confirmed that both roads were free of cavities.

#### 3.2.2. Vehicle-Induced Signal Extraction

**Figure 11:**
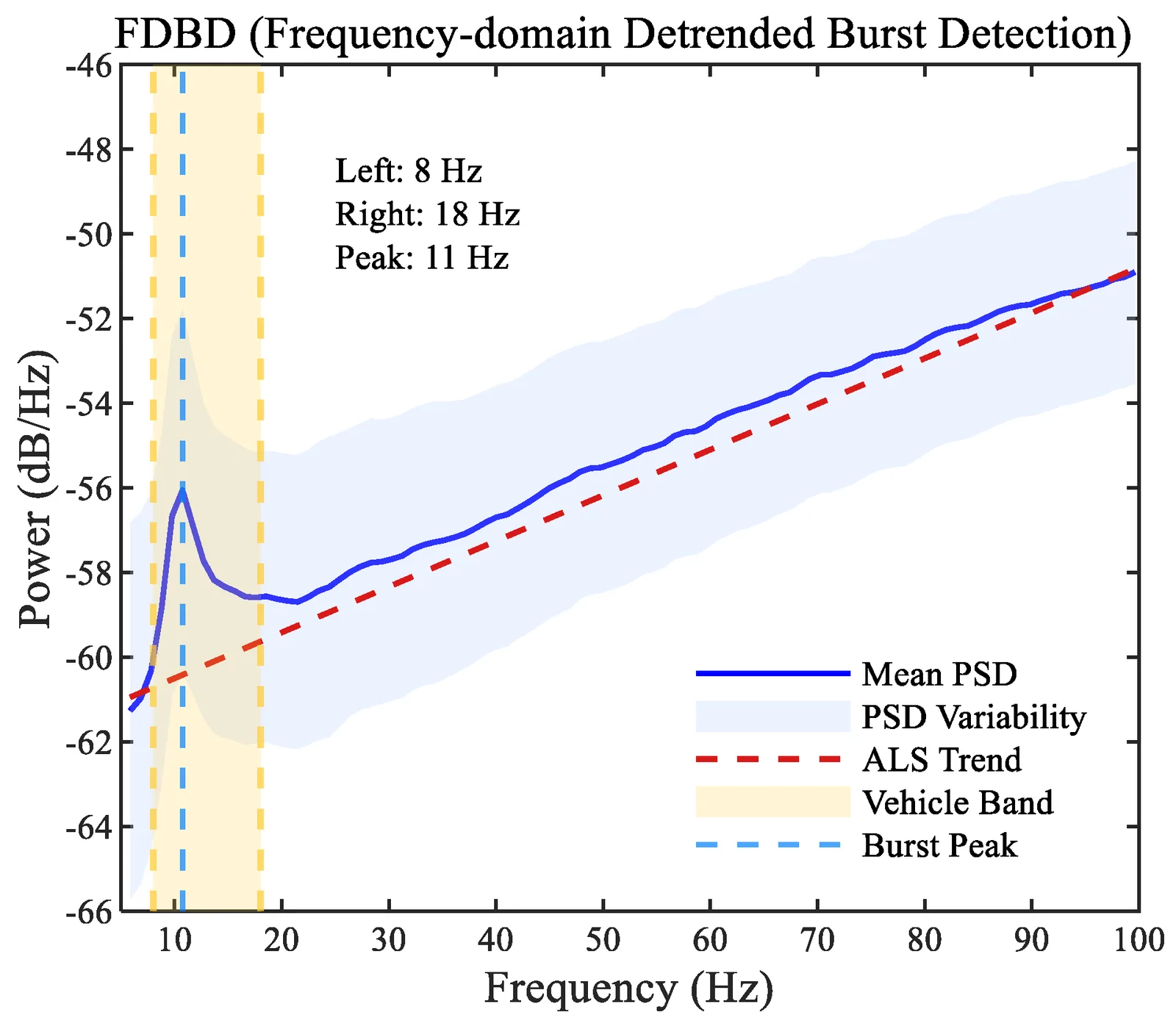
Frequency band selection for Road 1 using the FDBD method. The yellow shaded area represents the vehicle band, with yellow dashed lines marking its lower and upper frequency boundaries.

First, the frequency bands of vehicle-induced signals were selected using the FDBD method. Taking Road 1 as an example, the corresponding frequency band analysis is illustrated in Figure 11. Noticeable variations in the frequency bands were observed across different roads, mainly due to differences in traffic conditions and soil states during data collection. This observation highlights the need for the FDBD method to achieve adaptive frequency band selection.

**Figure 12:**
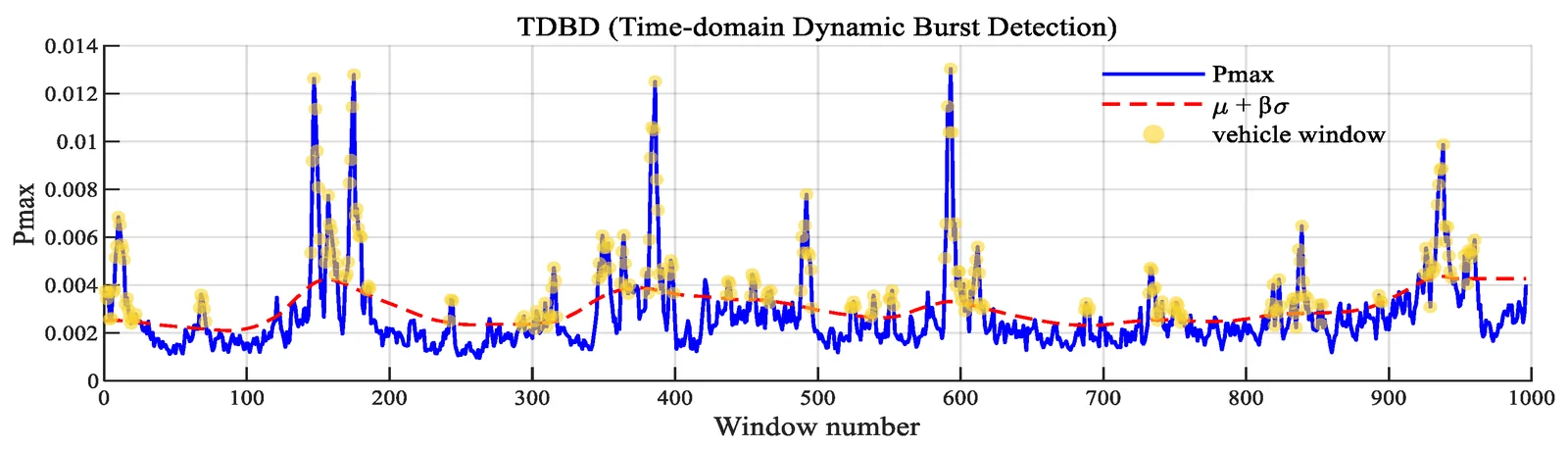
Vehicle-induced signal window selection for a single channel on Road 2 using the TDBD method.

On actual roads, vehicles are predominantly small cars, resulting in relatively lower spectral amplitude maxima of vehicle-induced signals. The distinction between these signals and background noise is less pronounced compared to experimental road conditions. Furthermore, the background noise in actual road vibration signals is considerably more complex than in experimental settings. As a result, the vehicle-induced signals are easily masked by noise. The high-amplitude windows become discontinuous, and isolated points increase, which together make signal identification and selection more challenging. Figure 12 presents the TDBD analysis results from a single channel of Road 2. To address noise fluctuations, TDBD employed a dynamic threshold strategy that automatically adjusts the threshold based on real-time noise levels to identify high-amplitude windows. Combined with connected-region analysis to remove isolated windows, this approach reduces false positives and improves the reliability of signal selection.

**Figure 13:**
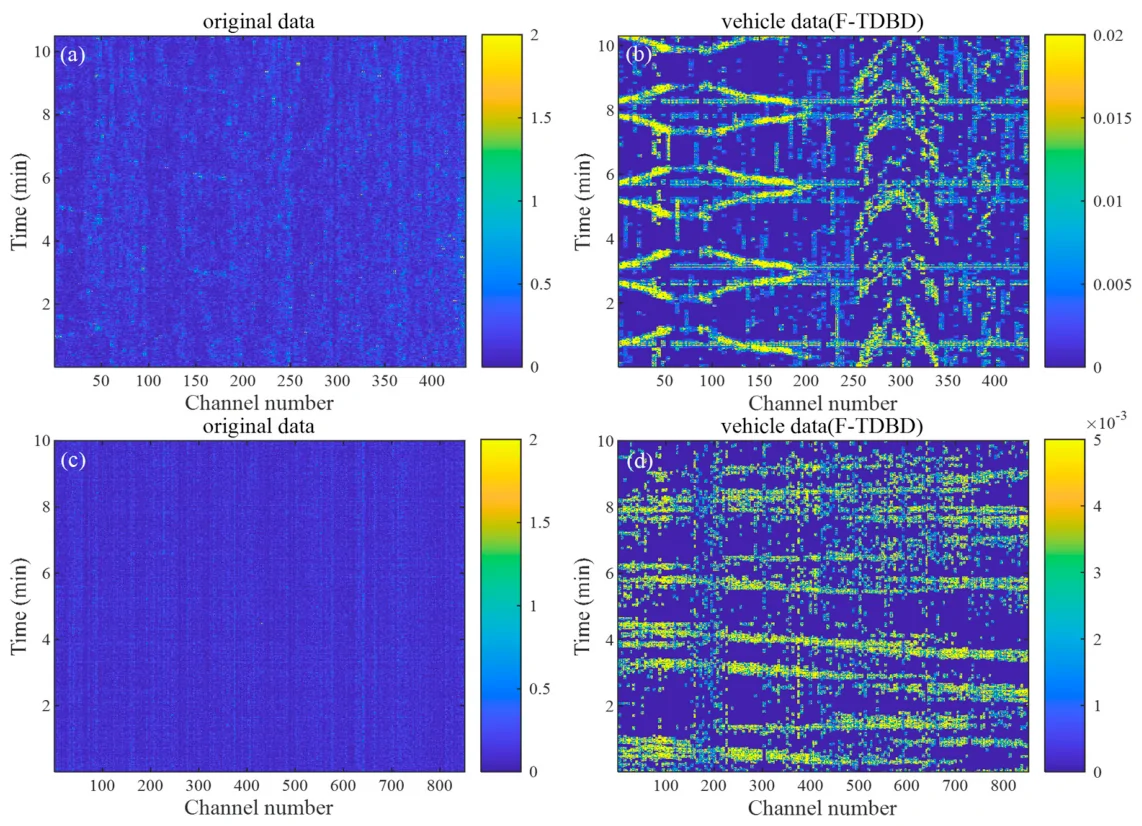
Spatiotemporal waterfall plots of the data for Roads 1 and 2 before and after FDBD and TDBD denoising: (**a**) Road 1 original data, (**b**) Road 1 vehicle-induced data, (**c**) Road 2 original data, (**d**) Road 2 vehicle-induced data.

Figure 13 shows 10 min waterfall plots before and after vehicle-induced signal extraction for both roads. After processing, background noise is greatly reduced, and the extracted vehicle-induced signals exhibit continuous trajectories consistent with expected physical patterns in both time and space.

#### 3.2.3. Field Road Test Results and Analysis

The optimal feature ratio set obtained from the experimental roads, together with its corresponding AFRI threshold, was applied to actual roads for stability evaluation. For each channel on the actual roads, the optimal feature ratio set was first computed, and the AFRI was then calculated across different data batches.

**Figure 14:**
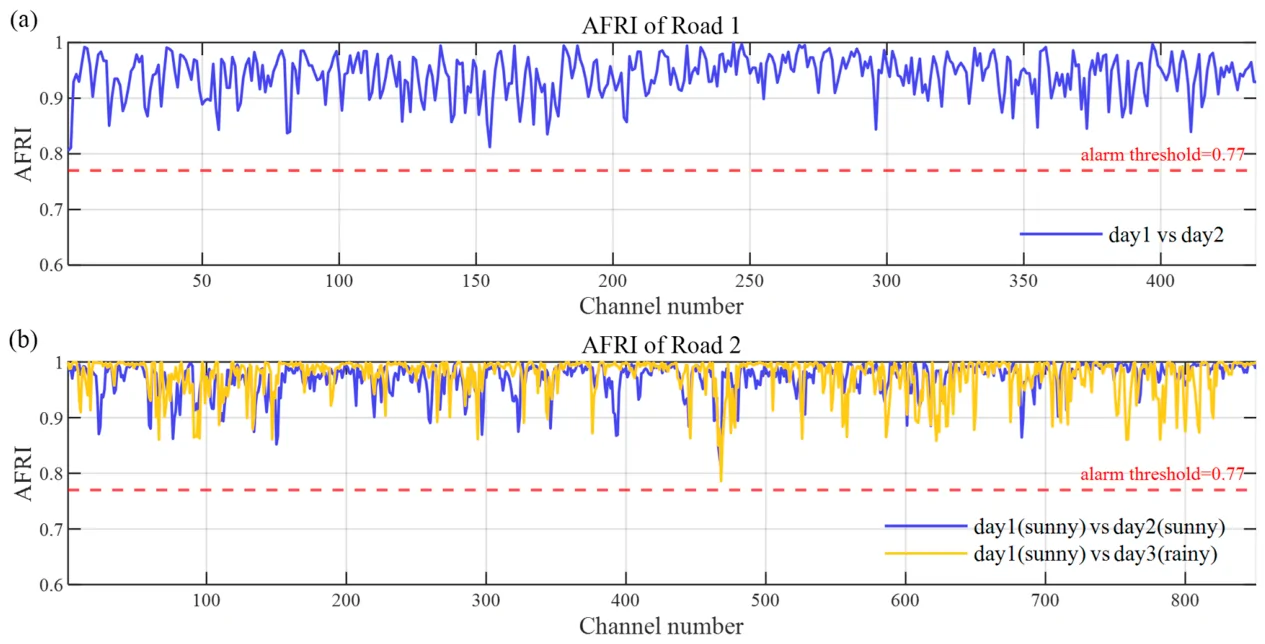
AFRI results for actual Roads 1 (**a**) and 2 (**b**).

As shown in Figure 14, the AFRI values on both roads remained above the empirical threshold, exhibiting limited and stable fluctuations under normal traffic and other non-cavity disturbances. No cavity anomalies were detected, which is consistent with the GPR inspection results. These findings demonstrate the stability of the proposed DAS-based early-warning method under complex traffic, weather, and urban noise conditions.

## 4. Discussion

The results support the central idea of this work: ordinary traffic, despite being intermittent and highly variable, can serve as a usable excitation source for detecting cavities beneath urban roads. The AFRI anomalies were spatially concentrated around the cavity, with the affected response extending to approximately 5 m from the cavity center. This value represents the observed spatial extent of the anomalous response rather than a formal localization error. The deviations also grew with the depth difference between compared batches, suggesting the indicator tracks the actual subsurface change rather than incidental disturbance. No controlled source, point-sensor array, or labeled data was required—the framework simply listens to vehicles already passing overhead.

Unlike earlier DAS-based cavity studies, which relied on active sources or strong, regular excitations such as trains and pipeline flows, this approach uses the traffic itself. The experiments show that this works only if extraction adapts to the prevailing noise: the dominant vehicle band varied between roads, and even between the sunny and rainy days of the Road 2 campaign, so a fixed band would likely have failed. Machine learning offers an alternative, but the annotation burden and poor transferability of trained models remain obstacles. Comparing each channel with itself through feature ratios removes much of the excitation variability and keeps the index physically interpretable—an advantage for unattended, long-term operation.

The frequency-domain observations also explain why spectral-shape features outperformed time-domain ones: the vehicle-dominated band shifted toward higher frequencies with increasing burial depth, and descriptors such as spectral kurtosis and skewness capture this directly, whereas amplitude-based metrics are more easily contaminated by day-to-day traffic variability. These field tests indicate that the index remained stable under the tested non-cavity conditions, although further validation on roads containing confirmed cavities is needed to assess its transferability.

Several limitations of this study should be noted. Data coverage remains limited, and further validation across diverse roads, traffic conditions, and subsurface structures is needed to improve generalizability. Response mechanisms of different void types, burial depths, and evolution processes require further investigation. Long-term stability is affected by fiber degradation and environmental variations. Future work could explore self-calibration and environment-adaptive strategies to enhance reliability. If these issues can be resolved, reuse of existing telecommunication fiber could support city-wide road health monitoring at a fraction of the cost of conventional surveys.

## 5. Conclusions

This study proposed a DAS-based monitoring framework that uses routine vehicle-induced vibrations to detect concealed cavities beneath urban roads. A time–frequency joint extraction algorithm was developed to isolate vehicle-induced responses under complex background noise, and the feature-ratio-based AFRI enabled unsupervised channel-level anomaly detection without active excitation or extensive labeled data.

Controlled experiments demonstrated the sensitivity of the proposed framework to cavities at different burial depths, while the field-road tests further demonstrated the stability of the index under different traffic and environmental conditions without false alarms. These results support the feasibility of using routine traffic and existing optical-fiber infrastructure for continuous monitoring of urban road subsurface conditions.

Nevertheless, the current validation is limited to controlled cavity conditions and a small number of cavity-free field roads. Further validation involving diverse road and subsurface conditions, traffic characteristics, and confirmed real-world cavities is required to establish the generalizability and long-term reliability of the proposed framework.

## Figures and Tables

**Table 1:** Independent frequency-domain feature metrics.

Feature Name	Symbol	Formula	Feature Name	Symbol	Formula
Mean	$F_{\mathrm{mean}}$	$\frac{1}{N}\sum_{i=1}^{N}X_{i}$	Impact Factor	$F_{\mathrm{IF}}$	$\frac{F_{\max}}{F_{\mathrm{mean}}}$
Minimum	$F_{\min}$	$\underset{1\leq i\leq N}{\min}X_{i}$	Margin Factor	$F_{\mathrm{MF}}$	$\frac{F_{\max}}{{\left(\frac{1}{N}\sum_{i=1}^{N}\sqrt{X_{i}}\right)}^{2}}$
Root Mean Square (RMS)	$F_{\mathrm{rms}}$	$\sqrt{\frac{1}{N}\sum_{i=1}^{N}X_{i}^{2}}$	Centroid Frequency	$F_{\mathrm{CF}}$	$\frac{\sum_{i=1}^{N}f_{i}X_{i}}{\sum_{i=1}^{N}X_{i}}$
Skewness	$F_{\mathrm{skew}}$	$\frac{\frac{1}{N}\sum_{i=1}^{N}{\left(X_{i}-F_{\mathrm{mean}}\right)}^{3}}{(\frac{1}{N}\sum_{i=1}^{N}{\left(X_{i}-F_{\mathrm{mean}}{)}^{2}\right)}^{3/2}}$	Mean-Square Frequency	$F_{\mathrm{MSF}}$	$\frac{\sum_{i=1}^{N}f_{i}^{2}X_{i}}{\sum_{i=1}^{N}X_{i}}$
Kurtosis	$F_{\mathrm{SK}}$	$\frac{\frac{1}{N}\sum_{i=1}^{N}{\left(X_{i}-F_{\mathrm{mean}}\right)}^{4}}{(\frac{1}{N}\sum_{i=1}^{N}{\left(X_{i}-F_{\mathrm{mean}}{)}^{2}\right)}^{2}}$	Frequency Variance	$F_{\mathrm{VF}}$	$\frac{\sum_{i=1}^{N}{\left(f_{i}-F_{\mathrm{CF}}\right)}^{2}X_{i}}{\sum_{i=1}^{N}X_{i}}$
Crest Factor	$F_{\mathrm{AF}}$	$\frac{F_{\max}}{F_{\mathrm{rms}}}$	Spectral Kurtosis–Mean	$F_{\text{SK\_mean}}$	$\frac{1}{N}\sum_{i=1}^{N}F_{\mathrm{SK},i}$
Waveform Factor	$F_{\mathrm{WF}}$	$\frac{F_{\mathrm{rms}}}{F_{\mathrm{mean}}}$	Spectral Kurtosis–Skewness	$F_{\text{SK\_skew}}$	$\frac{\sum_{i=1}^{N}{\left(F_{\mathrm{SK},i}-F_{\text{SK\_mean}}\right)}^{3}}{(\frac{1}{N}\sum_{i=1}^{N}{\left(F_{\mathrm{SK},i}-F_{\text{SK\_mean}}{)}^{2}\right)}^{3/2}}$

Note: $X_{i}$ denotes the amplitude at the i-th frequency bin; $f_{i}$ denotes the frequency of the i-th bin; N is the total number of frequency bins; $F_{\max}=\underset{1\leq i\leq N}{\max}X_{i}$ is the maximum spectral amplitude; $F_{\mathrm{SK},i}=\frac{\frac{1}{K}\sum_{j=1}^{K}{\left(X_{i,j}-\bar{X_{i}}\right)}^{4}}{(\frac{1}{K}\sum_{j=1}^{K}{\left(X_{i,j}-\bar{X_{i}}{)}^{2}\right)}^{2}}-3$ is the spectral kurtosis at the i-th frequency bin; where $\bar{X_{i}}=\frac{1}{K}\sum_{j=1}^{K}X_{i,j}$ $X_{i,j}$ represents the amplitude at the i-th frequency bin in the j-th time window w, and K is the total number of time windows.

**Table 2:** Independent time-domain feature metrics.

Feature Name	Symbol	Formula	Feature Name	Symbol	Formula
Mean	$T_{\mathrm{mean}}$	$\frac{1}{M}\sum_{i=1}^{M}x_{i}$	Root Mean Square (RMS)	$T_{\mathrm{rms}}$	$\sqrt{\frac{1}{M}\sum_{i=1}^{M}x_{i}^{2}}$
Maximum	$T_{\max}$	$\underset{1\leq i\leq M}{\max}x_{i}$	Skewness	$T_{\mathrm{skew}}$	$\frac{\frac{1}{M}\sum_{i=1}^{M}{\left(x_{i}-T_{\mathrm{mean}}\right)}^{3}}{{\left(\frac{1}{M}\sum_{i=1}^{M}{\left(x_{i}-T_{\mathrm{mean}}\right)}^{2}\right)}^{3/2}}$
Median	$T_{\mathrm{median}}$	$\mathrm{median}\left(x_{1},x_{2},\ldots ,x_{M}\right)$	Kurtosis	$T_{\mathrm{SK}}$	$\frac{\frac{1}{M}\sum_{i=1}^{M}{\left(x_{i}-T_{\mathrm{mean}}\right)}^{4}}{{\left(\frac{1}{M}\sum_{i=1}^{M}{\left(x_{i}-T_{\mathrm{mean}}\right)}^{2}\right)}^{2}}$

Note: $x_{i}$ denotes the amplitude of the i-th sample in the time domain; M is the length of the time-domain sequence.

## Data Availability

The data presented in this study are available on request from the corresponding author.

## Abbreviations

The following abbreviations are used in this manuscript:

AFRI	Average Feature Ratio Indicator
DAS	Distributed Acoustic Sensing
ERT	Electrical Resistivity Tomography
FDBD	Frequency-Domain Detrended Burst Detection
FPR	False-Positive Rate
GPR	Ground-Penetrating Radar
OFDR	Optical Frequency Domain Reflectometry
PVC	Polyvinyl Chloride
RMS	Root Mean Square
STFT	Short-Time Fourier Transform
TDBD	Time-Domain Dynamic Burst Detection
TEM	Transient Electromagnetic
